# Metformin Use and Survival in Patients with Advanced Extrahepatic Cholangiocarcinoma: A Single-Center Cohort Study in Fuyang, China

**DOI:** 10.1155/2021/9468227

**Published:** 2021-10-29

**Authors:** Jinqing Wu, Yabo Zhou, Guizhou Wang

**Affiliations:** Department of Gastroenterology, Fuyang People's Hospital, Fuyang 236000, China

## Abstract

**Aims:**

Metformin is an oral antidiabetic agent that has been widely prescribed for the treatment of type II diabetes. In recent years, anticancer properties of metformin have been revealed for numerous human malignancies. However, there are few indications available regarding the feasibility and safety of these studies in an advanced extrahepatic cholangiocarcinoma (EHCC) population. This study is aimed at evaluating the feasibility, safety, and value of metformin use and survival in patients with advanced EHCC.

**Methods:**

All patients with advanced EHCC observed at Fuyang People's Hospital between January 2015 and November 2020 were included in the study. Case data, clinical information, and imaging results were abstracted from the self-administered questionnaire and electronic medical record. All patients were divided into study subjects and control subjects, and the study subjects were given metformin, 0.5 g, three times a day, while control subjects were without metformin. The metformin use and survival time of the subjects were asked by telephone, out-patient, or door-to-door visit, after they left the hospital.

**Results:**

One hundred and thirty-three study cases and 589 controls were included in the analysis. This study showed that metformin use cannot improve the overall survival rate of patients with advanced EHCC ([95% CI]: -17.05-0.375, *t* = −1.889, *P* value = 0.061), but the survival time of patients with drainage treatment from control group (*n* = 496) was significantly shorter than that of patients with drainage treatment from the study group (*n* = 113), and the difference was statistically significant (*z* = −2.230, *P* value = 0.026). There were significant differences between metformin used before or after the diagnosis of advanced EHCC (OR[95% CI], 3.432[2.617-4.502]; *P* value = 0.001) in survival time. And there was significant difference between the duration of metformin use and survival prognosis (OR[95% CI], 2.967[1.383-6.368]; *P* = 0.005).

**Conclusion:**

Metformin can improve the survival of advanced EHCC patients who underwent drainage treatment, especially for metformin use after diagnosis of advanced EHCC and long duration of metformin.

## 1. Introduction

Cholangiocarcinoma (CCA) is categorized as an distal cholangiocarcinoma, perihilar, and intrahepatic [1]. CCA clinical presentation depends on the anatomic location and macroscopic growth pattern [1]. Early CCA often has no special clinical symptoms. Perihilar and distal CCA often have jaundice, which gradually deepens with time; light stool color; dark yellow urine color; and skin pruritus. Intrahepatic CCA is dominated by nonspecific symptoms like abdominal pain, fatigue, weight loss, night sweats, and cachexia; however, cirrhotic patients can be asymptomatic [1]. Histologically, 90% of CCA are adenocarcinomas, while known variants contain signet-ring type, papillary adenocarcinoma, squamous cell carcinoma, clear cell type, oat cell carcinoma, intestinal type adenocarcinoma, and adenosquamous carcinoma [1]. Surgery is the main curative method, whereas stent implantation by endoscopic retrograde cholangiopancreatography, percutaneous transhepatic cholangial drainage, systemic chemotherapy, and radiofrequency ablation are a curative option for advanced CCA [1, 2].CCA is a malignant tumor originating in the biliary tree, and it is the second most common cancer after hepatocellular carcinoma [2, 3]. The Bertuccio et al. study showed that mortality from extrahepatic (ECC) levelled off or decreased [4], while the EHCC appears to be one of the most rapidly increasing tumors in China [5, 6]. Although some treatment approaches were developed as therapeutics for EHCC, the prognosis of patients with unresectable or advanced EHCC is poor [7, 8]. More than 50% of cases with jaundice are inoperable at the time of first diagnosis [7].

Metformin is an oral antidiabetic agent that has been widespread prescribed for treatment of type II diabetes [9, 10]. This drug lowers hyperglycemia through the inhibition of hepatic glucose production. Compared to normal cells, cancer cells preferentially metabolize glucose to lactate, even in aerobic conditions. Such metabolic alterations not only promote the growth and invasion of tumor cells but also support their chemoresistance [11]. Kaewpitoon et al. suggested that metformin might influence tumorigenesis, both indirectly, through the systemic reduction of insulin levels, and directly, via the induction of energetic stress [12]. A recent epidemiologic survey indicated that metformin use was associated with reduced tumor incidence in patients with type II diabetes [13–16]. The anticarcinogenic activity of metformin has been attributed to many mechanisms, including the activation of the liver kinase B1 (LKB1)/AMP-activated protein kinase (AMPK) pathway, inhibition of the unfolded protein response, inhibition of protein synthesis, induction of cell cycle arrest and/or apoptosis, activation of the immune system, and potential eradication of cancer stem cells [12, 15, 17]. LKB1/AMPK pathway activation inhibits the mammalian target of rapamycin (mTOR), which negatively affects protein synthesis in tumor cells [15]. Several in vivo and in vitro studies have demonstrated metformin to inhibit the proliferation of various cancer cell types, including gastric, esophageal, breast, prostate, hepatocellular carcinoma, and colon cancer cells [12]. Anticancer properties of metformin have been revealed for numerous human malignancies including CCA with antiproliferative effects in vitro [3]. Moreover, some studies further found that metformin effectively sensitized CCA cells based on certain chemotherapies [18].

In this study, the effects of metformin on survival and prognosis of patients with advanced EHCC were assessed.

## 2. Materials and Methods

### 2.1. Study Population

All patients with advanced EHCC observed at Fuyang People's Hospital between January 2015 and November 2020 were included in the study. We searched for EHCC cases using an imaging diagnosis system and electronic medical record system. The diagnosis of advanced EHCC was confirmed by abdominal enhanced CT, abdominal enhanced MR, and PET-CT; all patients were confirmed to transfer (blood vessel, nerve, lymph gland and organ tissue). After screening, 722 patients with confirmed advanced EHCC were included in the analysis. They were divided into study subjects and control subjects, and the study subjects (*n* = 133) agreed to participate in the study and take metformin, 0.5 g, three times each day, which was produced by Guizhou Shengjitang Pharmaceutical Co., Ltd. Control subjects (*n* = 589) were without metformin. Cases were matched by sex, age, ethnicity, and residence to subjects who enrolled in the Fuyang People's Hospital between January 2015 and November 2020.This study was approved by the ethics committee of the Fuyang People's Hospital, China.

### 2.2. Clinical Information

Case data, clinical information, and imaging results were abstracted from the self-administered questionnaire and electronic medical record. Relevant factors abstracted included diabetes, history of liver disease (HBV or HCV infection), tumor location, radiotherapy, other drugs that may affect tumor (aspirin, regulating immunity, antitumor, and Chinese herbal medicine, etc.), drainage treatment, family history of tumor, drinking history, and smoking history.

We collected the results of tests for HBV and HCV infection for all subjects. HBV infection was defined as a positive hepatitis B surface antigen. And HCV infection was defined as positive HCV antibody or HCV RNA. We abstracted the results of tests for total bilirubin (TBil), transaminase (ALT and AST), bile duct enzyme (ALP and *γ*-GT), tumor index (AFP, CA199, and Hsp90*α*), jaundice, and ascites for all cases.

Previous or current use of metformin was ascertained from the questionnaire and physician's notes. And duration of metformin use was ascertained from the follow-up of patients or their families.

The subjects were given metformin, and the medication was recorded in detail; basing on the diagnosis of advanced EHCC was taken as the starting point of observation. The metformin use and survival time of the subjects were asked by telephone, out-patient, or door-to-door visit, after they leave the hospital. Six-month observation was regarded as the end point of the event, and the death was recorded as 0 and the survival was recorded as 1.

## 3. Statistical Analysis

*t*-test was used to compare the data of normal distribution among groups, and mean + SDwas used for expression. Mann–Whitney *U* test was used to analyze the difference between groups for nonnormal distribution data, and median (IQR) was used for skew distribution data. Pearson chi-square test (*χ*^2^) was used to analyze the differences between the groups. Odds ratios (OR) and 95% confidence intervals (95% CI) were used to estimate the relationship between metformin use and survival prognosis.

Kaplan-Meier survival analysis, log-rank tests, and Breslow were performed to analyze the overall survival (OS) of subjects. Box plot was used to compare and analyze the differences of different groups. Statistical analyses were done using SPSS software, version 20.0.All tests were two-sided or Fisher's exact test, with *P* < 0.05 defined as statistically significant.

## 4. Results

### 4.1. Patient Characteristics

One hundred and thirty-three study cases and 589 controls were included in the analysis. Tables 1 and 2 summarize the baseline characteristics and risk factors for the study and control groups that may affect the survival and prognosis. Demographics were comparable between groups. Trend analysis showed that there were no differences in baseline characteristics (*P* value > 0.05). And trend analysis showed that there were no differences in laboratory results (*P* value > 0.05).

### 4.2. Study Subject Characteristics

In the study group, 6 cases were treated with metformin before EHCC because of diabetes, while 127 cases were given metformin after EHCC. In the study group (*n* = 133), there was no significant difference between the age when metformin was started and survival time (OR[95% CI], 0.990[0.476-2.057]). However, there were significant differences between metformin used before or after the diagnosis of advanced EHCC (OR[95% CI], 3.432[2.617-4.502]; *P* value = 0.001) in survival time. There was significant difference between the duration of metformin use and survival prognosis (OR[95% CI], 2.967[1.383–6.368]; *P* value = 0.005) (Table 3).

### 4.3. Metformin Use Can Prolong the Survival Time of Patients Who Have Undergone Drainage Treatment

**Figure 1:**
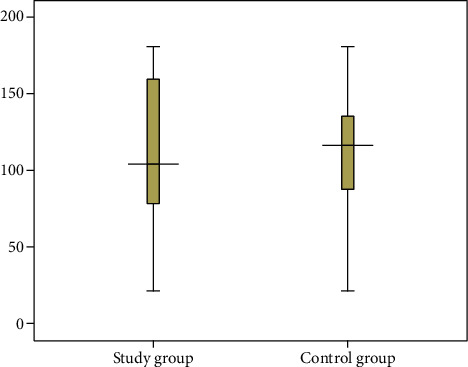
The box-plot distribution and comparative analysis of the survival time between overall study group (*n* = 133) and overall control group (*n* = 589), and no significant difference ([95% CI]: -17.05-0.375, *t* = −1.889, *P* value = 0.061).

**Figure 2:**
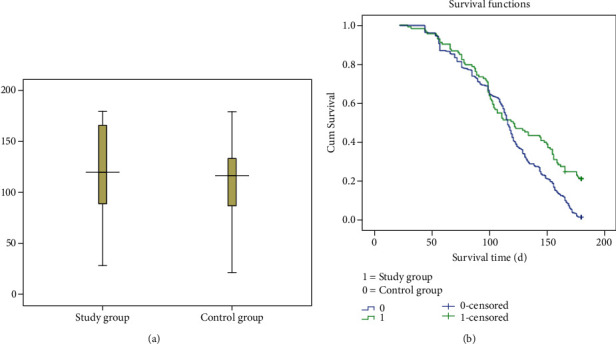
The box-plot distribution and comparative analysis of the survival time between patients with drainage treatment from study group (*n* = 113) and patients with drainage treatment from control group (*n* = 496) was different (*z* = −2.230, *P* value = 0.026). The Kaplan-Meier method was used to estimate survival rate and compare survival curve of patients with drainage treatment from two groups: the curve of control group was below, and the curve of study group was above, which showed that metformin use can improve survival rate. Log-rank tests and Breslow were performed to check; *P* value was less than 0.05.

**Figure 3:**
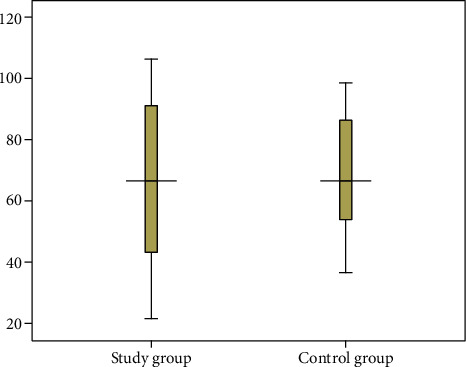
The box-plot distribution and comparative analysis survival time of patients without drainage treatment from study group (*n* = 20) and control group (*n* = 93) (65.0 ± 26.63 vs. 67.22 ± 22.16; [95% CI]: -9.012-13.442, *t* = 0.391, *P* value = 0.697).

Compared to the overall control group (*n* = 589), there was no significant difference in the survival time of the overall study group (*n* = 133) ([95% CI]: -17.05-0.375; *P* value = 0.061) (Figure 1). As expected, the survival time of patients with drainage treatment from the control group (*n* = 496) was significantly shorter than that of patients with drainage treatment from the study group (*n* = 113), and the difference was statistically significant (*z* = −2.230, *P* value = 0.026) (Table 4 and Figure 2). There was no significant difference in the survival time between patients without drainage treatment from the study group (*n* = 20) and patients without drainage treatment from the control group (*n* = 93) ([95% CI]: -9.012-13.442; *P* value = 0.697) (Figure 3). Compared to countryside patients, the survival time of town patients from the study group (*n* = 59) and the control group (*n* = 291) was significantly longer (101.03 ± 44.94 vs. 132.56 ± 44.59; 100.84 ± 41.27 vs. 112.66 ± 36.96), and the difference was statistically significant (*P* value < 0.01), Table 4.

### 4.4. The Value of Metformin Use in Feasibility and Safety

This study showed that the number of patients who actively withdrew from using metformin due to intolerance was only 7 (5.26%), while survival time was shorter (Table 5).

## 5. Discussion

EHCC is a highly aggressive epithelial malignancy and usually has a poor prognosis because of the insensitivity to therapies and difficulty in detection [19], particularly for advanced EHCC. The diagnosis of EHCC is very complex and usually requires a combination of clinical symptoms, endoscopic techniques, imaging techniques, and cytopathological tests. In recent years [3], metformin has received growing attention due to its promising anticancer potential observed in many human tumors. A number of epidemiologic studies showed that metformin use in patients with diabetes was associated with a decreased incidence of various cancers, including CCA, gastroenterological cancers, pancreatic cancer, and breast cancer [16]. To our knowledge, this is the first time to study the relationship between metformin use and the survival in advanced EHCC.

This study showed that metformin use cannot improve the overall survival rate of patients with advanced EHCC ([95% CI]: -17.05-0.375; *t* = −1.889, *P* value = 0.061, Figure 1), but the survival time of patients with drainage treatment using metformin was significantly longer than that of patients without metformin (*z* = −2.230, *P* value = 0.026). In recent decades, study showed that treatment with the antidiabetic drug metformin has been recently associated with decreased incidence of intrahepatic CCA. Metformin reverts the mesenchymal and epithelial-to-mesenchymal transition (EMT) traits in intrahepatic CCA by activating AMPK-FOXO3-related pathways suggesting it might have therapeutic implications [20]. Metformin treatment reverses EMT and downregulates the proteolytic enzyme matrix metalloproteinase (MMP-2), resulting in suppression of CCA cell migration and invasion. Some studies [10, 20] demonstrated that metformin exerted antitumoral effects by (1) inhibiting adenosine deaminase that converts AMP into IMP, resulting in AMP accumulation with a subsequent activation of AMPK; (2) activating AMPK that plays a role in cellular energy homeostasis [21]; (3) blocking the mitochondrial respiratory chain complex (NADH dehydrogenase) that impairs ATP synthesis and increasing the AMP/ATP ratio [15]; and (4) metformin targeting the AMPK/mTORC1 pathway in cholangiocarcinoma cells [9, 22]. Trinh et al. and Saengboonmee et al. [3, 23] studies showed that metformin exposure significantly reduced cancer cell proliferation, migration, and invasion [9], possibly involving the signal transducers and activators of the transcription 3(STAT3) pathway and nuclear factor-kappa B (NF-*κ*B) pathway and reversal of EMT marker expression. STAT3 plays important roles in cancer development and progression, and its expression was associated with shorter survival of patients with CCA. And they further suggest that metformin may be useful for CCA management.

Complexes of Cdk6 and Cdk4 with cyclin D1 are required for G1 phase progression [13]; however, complexes of Cdk2 with cyclin E are required for the G1 to S transition [21]. Metformin has been demonstrated to downregulate cyclin D1 in various tumor cell lines, including stomach, colon, liver, breast, and prostate cancer lines [12]. The findings shown here indicate that these major cell cycle regulators (Cdk4,cyclin D1, and phosphorylated Rb) may be intracellular targets of the metformin-mediated antiproliferative effect in people CCA cell lines. Metformin has been demonstrated to alter the phosphorylation of many proteins, including c-Src, *β*-catenin, CREB, Chk2, and Akt, in various cell lines. Fujimori et al. [13] findings indicate that metformin inhibits people CCA cell proliferation and cancer growth, potentially by suppressing cell cycle-related molecules through miRNA alterations. In the present work [15], Zhang et al. demonstrated that metformin treatment profoundly suppressed proliferations of two human CCA cell lines (QBC939 and MZ-CHA-1) in dose-dependent ways. Through comparing metformin-induced changes of metabolite levels between the CCA cells and normal HUVEC cells, they indicate that metformin profoundly aggravate the Warburg effect and promote glycolysis in CAA cells [11]. In the Tang et al. study, they found that metformin could suppress the Warburg effect in CCA, which promotes oxidative phosphorylation and decreases aerobic glycolysis, thus making CCA cells vulnerable to chemotherapy [11]. Moreover, metformin specifically increases UDP-GlcNAc and BCAAs, indicating the occurrence of autophagy and cell cycle arrest in metformin-treated CAA cells. Ling et al. showed that metformin sensitizes arsenic trioxide to suppress intrahepatic cholangiocarcinoma through the regulation of AMPK/p38 MAPK-ERK3/mTORC1 pathways [18]. Metformin altered the miRNA (mir124, 182, and 27b; let7b, 221, and 181a) expression to inhibit tumor proliferation [2].

This study showed that the patients who had used metformin before the diagnosis of advanced EHCC could not improve the survival, which may be related to advanced EHCC tolerance or noninsensitivity to metformin (Table 3). Compared to metformin used before the diagnosis of advanced EHCC, metformin used can significantly improve the survival after the diagnosis of advanced EHCC in the study group (OR[95% CI], 3.432[2.617–4.502]; *P* value = 0.001), which is related to the duration of use (OR[95% CI], 2.967[1.383–6.368]; *P* value = 0.005). Ling et al. studies showed that metformin exhibited a time-dependent and dose-dependent antiproliferation effect on intrahepatic cell lines, by mechanisms containing apoptosis induction and cell cycle arrest [9]. Metformin intake after starting chemotherapy can improve the clinical outcome in advanced cholangiocarcinomas [24]. Metformin could change the metabolic status of cancer cells and reverse the Warburg effect via the inhibition of lactate dehydrogenase A(LDHA), which was overexpressed in CCA tissues and indicated a shorter survival time [11]. However, a study showed that [25] the survival of forty-nine patients who continued taking metformin after CCA diagnosis was not different from that of one hundred and sixty-five patients never taking metformin (9.1 vs. 9.2 months; HR[95% CI], 0.8[0.6-1.2]; *P* value = 0.31). A history of any metformin use before CCA diagnosis (*n* = 79) also did not affect survival. So, metformin did not improve the survival of CCA patients with diabetes mellitus. Our study is consistent with that of Yang et al., but the data of the above study is too few, which needs multicenter verification.

This study also showed that the survival time of patients living in town is higher than patients living in the countryside, whether metformin is used or not, which may be related to the patients' cultural literacy, attention to the disease, scientific and effective modern medical intervention, and the patients' affordability.

In conclusion, we elucidated that metformin can improve the survival and prognosis of advanced EHCC patients who have undergone drainage treatment. Metformin is an inexpensive drug, and its use has been proven safe without severe adverse effects in people. Thus, our findings show that the use of metformin might be beneficial for advanced EHCC patients who have undergone drainage treatment and might be a potential therapeutic agent for the treatment of EHCC (Table 5).

## 6. Conclusion

In conclusion, this research article, for the first time, reports the use of metformin in advanced EHCC patients. The results demonstrate that metformin can improve the survival and prognosis of advanced EHCC patients who have undergone drainage treatment. It is a feasible, practical, and safe therapeutic agent for the treatment of advanced EHCC.

## Figures and Tables

**Table 1:** Baseline characteristics of the study and control groups [*n* (%)].

Characteristic	Study group	Control group	*χ* ^2^	*P* value
	*n* = 133	*n* = 589		
Sex [*n* (%)]				
Male	70 (52.6)	331 (56.2)	0.559	0.455
Female	63 (47.4)	258 (43.8)		
Age [*n* (%)]				
<60	59 (44.4)	249 (42.3)	0.193	0.660
≥60	74 (45.6)	340 (57.7)		
Smoking history [*n* (%)]				
Yes	23 (17.3)	96 (16.3)	0.078	0.780
No	110 (82.7)	493 (83.7)		
Drinking history [*n* (%)]				
Yes	22 (16.5)	90 (15.3)	0.132	0.717
No	111 (83.5)	499 (84.7)		
Diabetes [*n* (%)]				
Yes	6 (4.5)	29 (4.9)	0.040	0.842
No	127 (95.5)	560 (95.1)		
Hepatitis B or C history [*n* (%)]				
Yes	6 (4.5)	20 (3.4)	0.389	0.533
No	127 (95.5)	569 (96.6)		
Family history of tumor [*n* (%)]				
Yes	0 (0.0)	4 (0.07)	—	1.000
No	133 (100.0)	585 (99.93)		
Residence [*n* (%)]				
Town	59 (44.4)	291 (49.4)	1.106	0.293
Countryside	74 (45.6)	298 (50.6)		
Tumor location [*n* (%)]				
Perihilar CCA	39 (29.3)	181 (30.7)	0.045	0.833
Distal CCA	94 (70.7)	456 (69.3)		
Cholelithiasis [*n* (%)]				
Yes	0 (0.0)	11 (1.9)	1.431	0.232
No	133 (100.0)	578 (98.1)		
PSC or PBC [*n* (%)]				
Yes	0 (0.0)	1 (0.02)	—	1.000
No	133 (100.0)	588 (99.98)		
Jaundice [*n* (%)]				
Yes	126 (94.7)	532 (90.3)	2.617	0.106
No	7 (5.3)	57 (9.7)		
Ascites [*n* (%)]				
Yes	1 (0.08)	20 (3.4)	1.831	0.176
No	132 (99.92)	569 (96.6)		
Radiotherapy [*n* (%)]				
Yes	1 (0.08)	9 (1.5)	0.079	0.779
No	132 (99.92)	580 (98.5)		
Other drugs that may affect tumor [*n* (%)]^∗^				
Yes	0 (0.0)	16 (2.7)	—	0.053
No	133 (100.0)	573 (97.3)		
Drainage treatment [*n* (%)]^∗∗^				
Yes	113 (85.0)	496 (84.2)	0.046	0.829
No	20 (15.0)	93 (15.8)		

PSC: primary sclerosing cholangitis; PBC: primary biliary cholangitis. ^∗^Aspirin, immune regulation, antitumor, and Chinese herbal medicine, etc. ^∗∗^ERBD: endoscopic retrograde biliary drainage (ERBD); PTCD: percutaneous transhepatic cholangial drainage.

**Table 2:** Baseline characteristics of laboratory results (mean ± SD).

Characteristic	Study group	Control group	*t*-value	*P* value
	*n* = 133	*n* = 589		
TBil (*μ*mol/L)	177.05 ± 65.07	171.79 ± 64.54	-0.848	0.397
ALT (U/L)	387.65 ± 234.78	396.28 ± 231.04	0.388	0.698
AST (U/L)	386.67 ± 235.28	395.75 ± 230.83	0.408	0.683
ALP (U/L)	461.27 ± 231.23	486.83 ± 220.27	1.197	0.232
*γ*-GT (U/L)	452.10 ± 230.91	478.00 ± 219.25	1.219	0.223
PT (s)	48.23 ± 25.53	46.24 ± 25.09	-0.820	0.413
AFP (ng/ml)	405.48 ± 244.69	404.15 ± 235.65	-0.058	0.954
CA199 (kU/L)	317.67 ± 166.208	321.36 ± 161.20	0.237	0.813
Hsp90*α* (ng/mL)	106.47 ± 41.64	105.15 ± 40.45	-0.338	0.736

TBil: total bilirubin; ALT: alanine aminotransferase; AST: aspartate aminotransferase; ALP: alkaline phosphatase; *γ*-GT: *γ*-glutamyl transpeptidase; PT: prothrombin time; AFP: alpha fetoprotein; CA199: carbohydrate antigen199; Hsp90*α*: heat shock protein 90*α*.

**Table 3:** ORs and 95% CIs for metformin use in study group.

Characteristic	Survival time ≥ 3 mon	Survival time < 3 mon	OR (95% CI)	*P* value
	*n* = 90	*n* = 43		
Age at first metformin use (y)				
<60	40	19	0.990 (0.476–2.057)	0.978
≥60	50	24		
Before or after EHCC use				
Before	0	6	3.432 (2.617–4.502)	0.001
After	90	37		
Duration of use (mon)				
<3	37	29	2.967 (1.383–6.368)	0.005
≥3	53	14		

*n*: number; y: year; d: day; mon: month.

**Table 4:** Comparison of survival time according to different characteristics grouping.

Group	*n*	Survival time (d)	*z*/*t*-value	*P* value
		Median (IQR)/(mean + SD)		
Drainage treatment^∗^				
Study group	113	121.0 (89.0,166.0)	-2.230	0.026
Control group	496	116.0 (85.0,144.0)		
No special treatment				
Study group	20	65.0 ± 26.63	0.391	0.697
Control group	93	67.22 ± 22.16		
Study group				
Town	59	132.56 ± 44.59	-4.034	≤0.001
Countryside	74	101.03 ± 44.94		
Control group				
Town	291	112.66 ± 36.96	-3.665	≤0.001
Countryside	298	100.84 ± 41.27		

^∗^Endoscopic retrograde biliary drainage (ERBD) or percutaneous transhepatic cholangial drainage (PTCD).

**Table 5:** Complications during metformin use in the study group.

	*N*	Metformin used time	Survival time
		Median (IQR) (d)	Median (IQR) (d)
Tolerable	126	106.00 (80.50,160.75)	106.50 (80.50,163.00)
Active withdrawal due to intolerance	7	30.00 (14.00,60.00)	73.00 (32.00,95.00)
*P* value		≤0.001	0.014

## Data Availability

The data used to support the findings of this study are available from the corresponding author upon request.

## References

[1] Brandi G., Venturi M., Pantaleo M. A. (2016). Cholangiocarcinoma: current opinion on clinical practice diagnostic and therapeutic algorithms: a review of the literature and a long-standing experience of a referral center. *Digestive and Liver Disease*.

[2] Neuzillet C., Casadei Gardini A., Brieau B. (2019). Prediction of survival with second-line therapy in biliary tract cancer: actualisation of the AGEO CT2BIL cohort and European multicentre validations. *European Journal of Cancer*.

[3] Trinh S. X., Nguyen H. T., Saimuang K., Prachayasittikul V., On W. C. (2017). Metformin inhibits migration and invasion of cholangiocarcinoma cells. *Asian Pacific Journal of Cancer Prevention*.

[4] Bertuccio P., Malvezzi M., Carioli G. (2019). Global trends in mortality from intrahepatic and extrahepatic cholangiocarcinoma. *Journal of Hepatology*.

[5] Hsing A. W., Gao Y. T., Devesa S. S., Jin F., Fraumeni J. F. (1998). Rising incidence of biliary tract cancers in Shanghai, China. *International Journal of Cancer*.

[6] Bao P. P., Zheng Y., Wu C. X. (2016). Cancer incidence in urban Shanghai, 1973-2010: an updated trend and age-period-cohort effects. *BMC Cancer*.

[7] Tantau A. I., Mandrutiu A., Pop A. (2021). Extrahepatic cholangiocarcinoma: current status of endoscopic approach and additional therapies. *World Journal of Hepatology*.

[8] Cadamuro M., Lasagni A., Lamarca A. (2021). Targeted therapies for extrahepatic cholangiocarcinoma: preclinical and clinical development and prospects for the clinic. *Expert Opinion on Investigational Drugs*.

[9] Ling S., Feng T., Ke Q. (2014). Metformin inhibits proliferation and enhances chemosensitivity of intrahepatic cholangiocarcinoma cell lines. *Oncology Reports*.

[10] Bhat A., Sebastiani G., Bhat M. (2015). Systematic review: preventive and therapeutic applications of metformin in liver disease. *World Journal of Hepatology*.

[11] Tang D., Xu L., Zhang M. (2018). Metformin facilitates BG45-induced apoptosis via an anti-Warburg effect in cholangiocarcinoma cells. *Oncology Reports*.

[12] Kaewpitoon S. J., Loyd R. A., Rujirakul R. (2015). Benefits of metformin use for cholangiocarcinoma. *Asian Pacific Journal of Cancer Prevention*.

[13] Fujimori T., Kato K., Fujihara S. (2015). Antitumor effect of metformin on cholangiocarcinoma: in vitro and in vivo studies. *Oncology Reports*.

[14] Landman G. W., Kleefstra N., van Hateren K. J., Groenier K. H., Gans R. O. B., Bilo H. J. G. (2010). Metformin associated with lower cancer mortality in type 2 diabetes: zODIAC-16. *Diabetes Care*.

[15] Zhang J., Hang C., Jiang T. (2020). Nuclear magnetic resonance-based metabolomic analysis of the anticancer effect of metformin treatment on cholangiocarcinoma cells. *Frontiers in Oncology*.

[16] Sookaromdee P., Wiwanitkit V. (2020). Decreased risk of cholangiocarcinoma in diabetic patients treated with metformin. *Journal of Cancer Research and Therapeutics*.

[17] Wandee J., Prawan A., Senggunprai L., Kongpetch S., Tusskorn O., Kukongviriyapan V. (2018). Metformin enhances cisplatin induced inhibition of cholangiocarcinoma cells via AMPK-mTOR pathway. *Life Sciences*.

[18] Ling S., Xie H., Yang F. (2017). Metformin potentiates the effect of arsenic trioxide suppressing intrahepatic cholangiocarcinoma: roles of p 38 MAPK, ERK3, and mTORC1. *Journal of Hematology & Oncology*.

[19] Li J., Yang Z., Huang S., Li D. (2020). BIRC7 and STC2 expression are associated with tumorigenesis and poor outcome in extrahepatic cholangiocarcinoma. *Technology in Cancer Research & Treatment*.

[20] di Matteo S., Nevi L., Overi D. (2021). Metformin exerts anti-cancerogenic effects and reverses epithelial-to- mesenchymal transition trait in primary human intrahepatic cholangiocarcinoma cells. *Scientific Reports*.

[21] Zhu H. Q., Ma J. B., Song X. (2016). Metformin potentiates the anticancer activities of gemcitabine and cisplatin against cholangiocarcinoma cells in vitro and in vivo. *Oncology Reports*.

[22] Bhat M., Sonenberg N., Gores G. J. (2013). The mTOR pathway in hepatic malignancies. *Hepatology*.

[23] Saengboonmee C., Seubwai W., Cha’on U., Sawanyawisuth K., Wongkham S., Wongkham C. (2017). Metformin exerts antiproliferative and anti-metastatic effects against cholangiocarcinoma cells by targeting STAT3 and NF-ĸB. *Anticancer Research*.

[24] Casadei-Gardini A., Filippi R., Rimini M. (2021). Effects of metformin and vitamin D on clinical outcome in cholangiocarcinoma patients. *Oncology*.

[25] Yang Z., Zhang X., Roberts R. O., Roberts L. R., Chaiteerakij R. (2016). Metformin does not improve survival of cholangiocarcinoma patients with diabetes. *Hepatology*.

